# Supplementary material for: Estimating total human deaths from rabies: A statistical framework leveraging reports of rabies transmission from deceased organ and tissue donors
Source: PLoS Negl Trop Dis. 2026 Sep 21;20(9):e0014731. doi: 10.1371/journal.pntd.0014731 (PMC13592719; doi:10.1371/journal.pntd.0014731)
Supplement: Supplementary file 6 [file pntd.0014731.s005.zip › Result_A/Malaysia/Figure_S3_Malaysia.pdf]

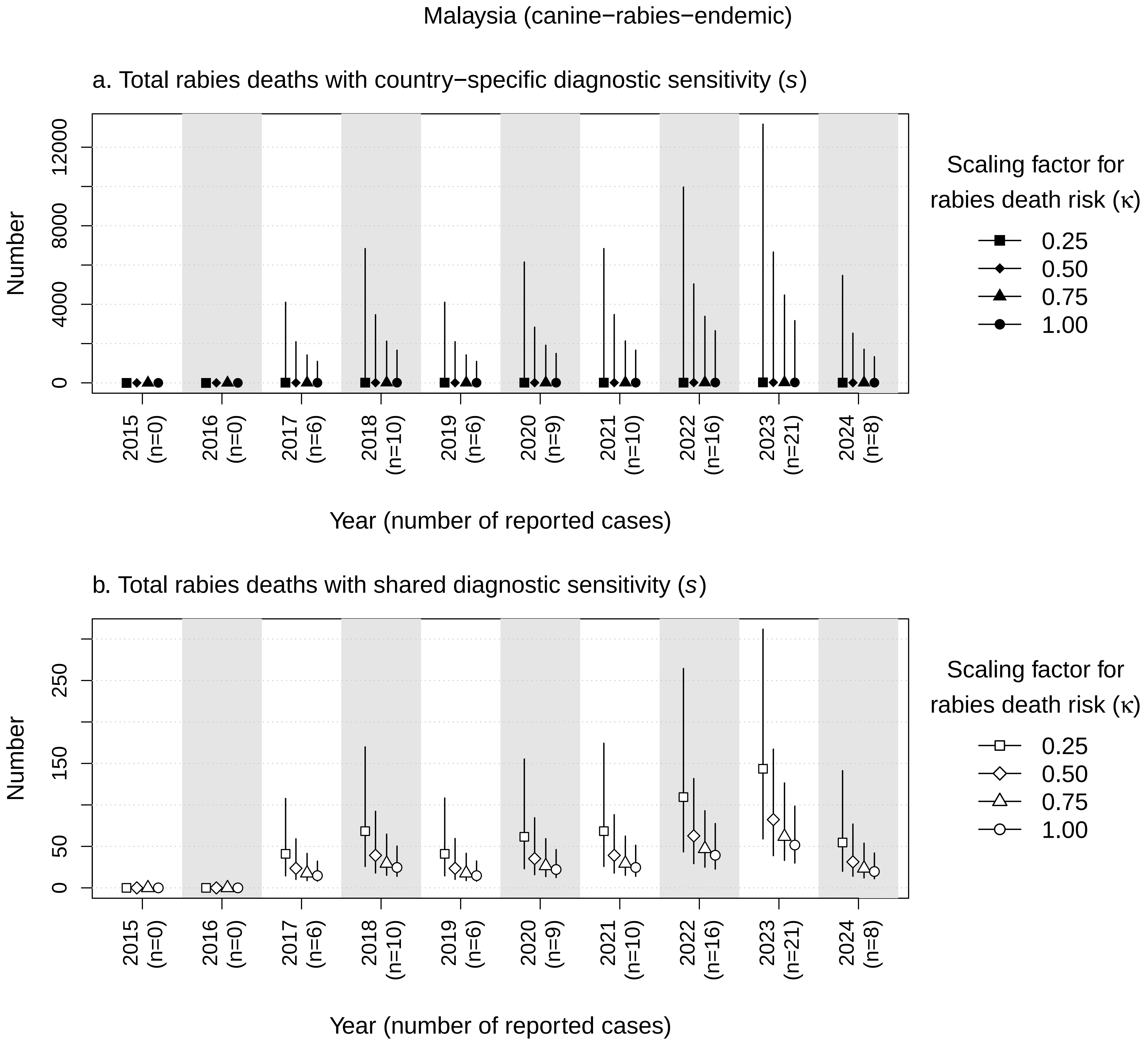

Malaysia (canine−rabies−endemic)
a. Total rabies deaths with country−specific diagnostic sensitivity (s)
Number
0
4000
8000
12000
2015 (n=0)
2016 (n=0)
2017 (n=6)
2018 (n=10)
2019 (n=6)
2020 (n=9)
2021 (n=10)
2022 (n=16)
2023 (n=21)
2024 (n=8)
Year (number of reported cases)
Scaling factor for rabies death risk (κ)
0.25
0.50
0.75
1.00
b. Total rabies deaths with shared diagnostic sensitivity (s)
Number
0
50
150
250
2015 (n=0)
2016 (n=0)
2017 (n=6)
2018 (n=10)
2019 (n=6)
2020 (n=9)
2021 (n=10)
2022 (n=16)
2023 (n=21)
2024 (n=8)
Year (number of reported cases)
Scaling factor for rabies death risk (κ)
0.25
0.50
0.75
1.00
